# Discovery of PACAP and its receptors in the brain

**DOI:** 10.1186/s10194-018-0855-1

**Published:** 2018-04-04

**Authors:** Takahiro Hirabayashi, Tomoya Nakamachi, Seiji Shioda

**Affiliations:** 1 Peptide Drug Innovation, Global Research Center for Innovative Life Science, Hoshi University, Ebara, Shinagawa-ku, Tokyo, 142-8501 Japan; 2 Laboratory of Regulatory Biology, Graduate School of Science and Engineering, University of Toyama, 3190-Gofuku, Toyama-shi, Toyama, 930-8555 Japan

**Keywords:** Neuropeptide, PACAP, PAC1-R, VPAC1-R, VPAC2-R, G protein-coupled receptors, Molecular cloning

## Abstract

Pituitary adenylate-cyclase-activating polypeptide (PACAP) is a 27- or 38-amino acid neuropeptide, which belongs to the vasoactive intestinal polypeptide (VIP)/glucagon/secretin family. PACAP shows particularly high homology (~ 68%) to VIP. Because of the high homology of the amino acid sequences of PACAP and VIP, these peptides share three class B-G-protein coupled receptors: the PAC1-Receptor (PAC1-R), the VPAC1-Receptor (VPAC1-R) and VPAC2-Receptor (VPAC2-R). These receptors have high homology to each other, and their high homology is utilized for these discoveries. This review provides mainly an overview of the history of the discovery of PACAP and its three receptors.

## Review

### Introduction

Pituitary adenylate-cyclase-activating polypeptide (PACAP) is a 27- or 38-amino acid neuropeptide, which shows particularly high homology to vasoactive intestinal peptide (VIP). These peptides share two common G protein-coupled receptors (GPCRs), VPAC1-R and VPAC2-R, while PACAP also has an additional specific receptor, PAC1-R. PACAP is primarily expressed in nervous tissues, where its receptors are also widely distributed.

PACAP and its receptors are involved in diverse biological functions. Peripherally, they function in the control of anterior pituitary hormone secretion, vasodilation, adrenaline secretion, insulin secretion and immunosuppression [1]. In the central nervous system, in addition to acting as a neurotransmitter, PACAP exerts a neuroprotective effect in response to cerebral brain ischemia, Parkinson’s disease, traumatic brain injury and spinal injury [2].

More recently, we have reported that PACAP gene-deficient mice develop dry eye-like symptoms such as corneal keratinization and tear reduction, and have shown that PACAP eye drops stimulate tear secretion via an adenylyl cyclase/ cyclic adenylyl cyclase monophosphate/protein kinase A (AC/cAMP/PKA) cascade, which in turn stimulates the translocation of aquaporin 5 from the cytosol to the membrane of lacrimal acinar cells to bring about an increase in water permeability [3]. It has also been demonstrated that injection of PACAP into the footpads of mice significantly promotes sweat secretion at the injection site [4]. These reports suggest that PACAP could also prove clinically useful for the treatment of dry eye disorder and sweating disorder.

A historical table of important discoveries related to effects of PACAP and its receptors in CNS is shown in Table 1.

**Table 1:** A historical table of important discoveries related to effects of PACAP and its receptors

Year	First discovery in animals	First discovery in humans	Important discoveries related to PACAP and its receptors in CNS
1989	Isolation of PACAP38 from an ovine hypothalamus extract [13]		stimulation of cAMP production [13]
1990	Isolation of PACAP27 from an ovine hypothalamus extract [14]	Cloning of human precursor of PACAP cDNA [11]	
	Cloning of ovine precursor of PACAP cDNA [11]		
1992	Cloning of rat VPAC1R [27]		Reduction of food uptake [53]
1993	Cloning of rat PAC1R cDNA [20]	Cloning of human PAC1R cDNA [25]	Increase in vasopressin release [54]
	Cloning of rat VPAC2R cDNA [23]	Cloning of human VPAC1R cDNA [31]	
1995			Stimulation of drinking [55]
1996			Increase in gonadotropin-releasing hormone (GnRH), somatostatin [56]
			Inhibition of luteinizing hormone secretion [57]
			Inhibition of apoptosis in neurons [58, 59]
			Suppression of ischemia-induced death of hippocampal neurons [60]
1998			Involvement in the circadian pacemaker clock [61]
2000			Generation of PAC1-R KO mice [62]
			Improvement of the learning and memory processes in a passive avoidance paradigm [63]
2001			Generation of PACAP KO mice [64, 65]
2003			Proliferation in astrocytes [66]
2004			Neuronal differentiation of mouse ES cells [67]
			Association of PACAP with retardation, phychotic behavior, hyperactive behavior [68]
2006			Neuronal differentiation of neural progenitor cells [69]
2007			Association of PACAP and PAC1-R with schizophrenia [70]
2010			Association of PACAP with major depressive disorder [71]
2011			Association of PAC1-R with PTSD [72]
2013			Association of VPAC2-R with schizophrenia [73]
2014			Suppression of cortical damage with traumatic brain injury [74]

In this article we provide an overview of the history of the discovery of PACAP and its three receptors.

### Overview of PACAP and its receptors

PACAP exists in two forms: PACAP38, which consists of 38 amino acids and PACAP27 which contains 27 amino acids at its N-terminus. These are produced by alternate processing from the PACAP precursor (prepro-PACAP), as discussed below. PACAP belongs to the VIP/glucagon/secretin family, sharing 68% homology of its amino acid sequence with VIP and 37% with secretin. Glucagon-like peptide (GLP)-1, GLP-2, growth hormone secretion hormone (GHRH), glucose-dependent insulinotropic polypeptide (GIP), and peptide histidine-methionine (PHM), which is attracting attention as a potent anti-diabetic drug, also belong to this family. Because of the high homology of the amino acid sequences of PACAP and VIP, these peptides share three class B-G-protein coupled receptors: the PAC1 receptor (PAC1-R), VPAC1 receptor (PVAC1-R) and VPAC2 receptor (VPAC2-R).

PACAP binding sites were studied using a radioreceptor assay with ^125^I-labelled PACAP in various tissues. The PACAP specific binding sites which were not shared VIP were termed type I, while the binding sites shared with both PACAP and VIP were termed Type II. TypeI binding sites show high affinity for PACAP38 and PACAP27 (Kd = approximately 0.5 nM) and much lower affinity for VIP (Kd > 500 nM) [5–8]. Type I binding sites are subdivided into the two subtypes named type IA and type IB. Type IA binding sites has an almost equal affinity for PACAP38, whereas type IB binding sites has a considerably greater affinity for PACAP38 than for PACAP27 [9]. Type II binding sites exhibit similar affinity for PACAP and VIP (Kd = approximately 0.5 nM) [6, 7].

According to the affinity for PACAP and VIP, the PAC1-R is classified into type 1 receptor, and VPAC1-R and VPAC2-R are classified into the type II, respectively.

**Fig. 1:**
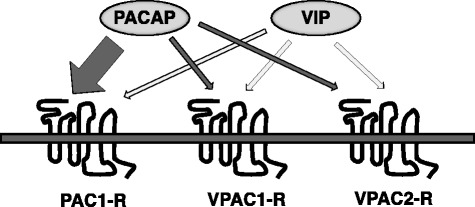
Comparison of the receptor affinity of PACAP and VIP. PACAP shows a > 1000-times higher affinity for PAC1-R than VPAC1-R or VPAC2-R [5–8]

Thus, VPAC1-R and VPAC2-R have comparable affinity for PACAP38, PACAP27 and VIP, whereas the affinity of PAC1-R for PACAP38 and PACAP27 is at least 1000 times greater than that for VIP (Fig. 1).

These receptors are associated with AC through cAMP, and activate PKA, which in turn can activate the mitogen-activated protein kinase (MAPK) pathway. PAC1-R is also coupled to phospholipase C, the activation of which stimulates Ca^2+^ mobilization and protein kinase C activation [10].

### Discovery of PACAP

In the 1980s, many of the hypothalamic releasing hormones that had been isolated, including growth hormone releasing hormone (GHRH) and corticotropin releasing hormone (CRH), had been reported to stimulate pituitary AC and increase cAMP accumulation [11, 12]. To isolate novel hypophysiotropic neuropeptides, Miyata, Arimura and colleagues screened fractions from an extract of ovine hypothalamic tissue (4370 pieces; 2400 g) by monitoring their AC activity in cultured rat anterior pituitary cells. Based on this approach, they isolated a novel neuropeptide comprising 38 amino acid residues. This peptide was named PACAP38 [13], which, based on the homology (68%) of its N-terminus amino acid sequence (amino acids 1-28) with that of ovine VIP, a 28 amino acid peptide hormone, was considered to belong to the VIP/glucagon/secretin family.

As the amino acid sequence of PACAP38 has a cleavage-amidation site (Gly28-Arg29-Arg30), it was anticipated that it would be possible to generate a shorter form of the peptide. Less than a year later, this proved to be the case when PACAP27, a C-terminally truncated form of PACAP38 with 27 residues was isolated. The shorter peptide was shown to display similar biological activity in terms of AC stimulation to PACAP38, at a level about 1000 times greater than that of VIP [14]. The amino acid sequence of PACAP is identical in all mammals, and in species such as the chicken, frog, salmon, and tunicate, only a few amino acids are substituted. This suggests that PACAP is highly conserved and has remained almost unchanged during an evolutionary period of ∼700 million years, indicating that it must play an important role in physiological function [15].

**Fig. 2:**
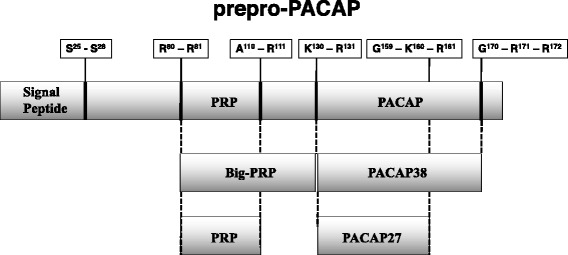
Structure of human prepro-PACAP. PACAP38 and PACAP27 are produced by alternative processing from the PACAP precursor termed prepro-PACAP [18, 19]

At around the same time, in 1990, the cDNA encoding the precursor of PACAP38 was successfully cloned from an ovine hypothalamus cDNA library by Kimura and colleagues [16]. In this study, they used a chemically synthesized oligonucleotide coding the 1-27 amino acid sequence of PACAP38 as a probe, together with a cDNA-encoded precursor of human PACAP38 from a human testis cDNA library, using a synthetic oligonucleotide probe [16]. Another group subsequently reported cloning of the precursor of human PACAP38 and the human PACAP gene [17]. The former cDNA clone encodes a protein of 176 amino acids, currently known as “prepro-PACAP”. Prepro-PACAP (amino acids 1-176) is initially metabolized by signal proteases to generate the signal peptide (amino acids 1-25) and pro-PACAP (amino acids 26-176). Pro-PACAP is metabolized by pro-hormone convertases and carboxypeptidases to produce a small fragment (amino acids 26-79), a large PACAP-related peptide (amino acids 82-129; PRP, the physiological function of which remains unclear) and C-terminal peptides (amino acids 132-170). The C-terminal peptides (amino acids 132-170 and 132-159) are metabolized by peptidylglycine alpha-amidating monooxygenase enzymes to PACAP38 and PACAP27, respectively, which have amidated C-terminals [18, 19] (Fig. 2). In the case of PACAP 27, analysis of the genomic structure of human PACAP has revealed that it is not generated by alternative splicing mechanisms, and PACAP precursor splice variants have not been reported.

### Discovery of PAC1-R

In 1993, Pisegna and Wank reported the cloning of a PACAP-selective receptor termed PACAP type I receptor (PAC1-R) which encodes a 495-amino acid protein with seven putative transmembrane domains and shows high homology with the VIP receptor, secretin receptor, GLP-1, PTH-PTHrP (parathyroid hormone and parathyroid hormone-related peptide) receptor, and calcitonin receptor. This novel receptor was screened using a rat VIP receptor cDNA as a probe by cross-hybridization from a cDNA library which was constructed from the rat pancreatic acinar carcinoma cell line AR4-2 J. The results of a binding assay with ^125^I-labeled PACAP and stimulation of cellular cAMP accumulation by PACAP in COS-7 cells transfected with the novel receptor cDNA were characteristic of a PACAP-specific receptor [20].

Within a short period, several other groups independently reported the existence of this receptor. For example, Hashimoto and colleagues isolated it as a PACAP receptor from a rat brain cDNA library by cross-hybridization with rat VIP receptor cDNA under low stringency conditions [21]. Using a similar approach, Hosoya and colleagues also cloned the receptor [22], as did Morrow and colleagues based on RT-PCR. The latter group first performed RT-PCR using rat anterior pituitary gland RNA as a template with a pair of degenerate oligonucleotide primers, corresponding to conserved regions in the third and seventh transmembrane domains of the rat secretin, pig calcitonin, and opossum PTH receptors. They then screened a rat olfactory bulb cDNA library with one of the PCR products as the probe, and isolated the receptor [23]. This receptor was termed the PAC1-Receptor by the International Union of Pharmacology [24] (Table 2).

**Table 2:** Nomenclature of PACAP and VIP receptors by IUPHAR

IUPHAR nomenclature	Gene symbol (HGNC)	Gene name (HGNC)	Previous nomenclature
PAC1	ADCYAP1R1	ADCYAP receptor type I	PACAP typeI receptor [20]
			PACAP receptor [21]
VPAC1	VIPR1	vasoactive intestinal peptide receptor 1	VIP receptor [27]
			VIP-PACAP typeII receptor [29]
			PVR2 [30]
VPAC2	VIPR2	vasoactive intestinal peptide receptor 2	VIP2 receptor [28]
			PVR3 [30]
			PACAPR-3 [32]

Three PACAP receptors have been classified according to their relative affinity for PACAP and VIP

In human, PAC1-Receptor was isolated from a human pituitary cDNA library. and the amino acid sequences of the human PAC1-R shows 92.5% homology with that of rat PAC1-R protein [25].

### Discovery of VPAC1-R

In 1991, Ishihara and colleagues isolated the rat secretin receptor based on a direct expression cloning strategy. In this method, a CDM8 expression library from the cDNA of NG108-15 cells, a hybrid cell line of mouse N18TG2 neuroblastoma and rat C6Bu-1 glioma, was transfected into COS cells, and the cells were assayed for their ability to bind secretin [26]. In the following year, using the secretin receptor cDNA as a probe, the same group cloned a novel receptor which encoded a 459-amino acid protein for VIP from a rat lung cDNA library by cross-hybridization. This novel receptor, which exhibited 50% amino acid sequence identity with rat PAC1-R, was initially designated the VIP receptor [27].

Subsequently, Lutz and colleagues also cloned the receptor using the same methods employed in the isolation of PAC1-R [23], but termed it the VIP1 receptor [28]. Reports of this novel receptor continued thereafter, variously referring to it as the VIP-PACAP type II receptor [29], or PVR2 [30].

However it has now been termed the VPAC1-Receptor by the International Union of Pharmacology [24] (Table 2).

The human VPAC1-R has been cloned from a HT29 human colonic adenocarcinoma cell line cDNA library using rat secretin cDNA sequence as a probe. The human VPAC1-R comprises 457 amino acids, and human and rat VPAC2-R proteins ehhibt 84% amino acids identity [31].

### Discovery of VPAC2-R

In 1993, Lutz and colleagues also cloned another novel VIP receptor which they referred to as the VIP2 receptor. First, they isolated a cDNA fragment from rat pituitary gland mRNA by RT-PCR using degenerate oligonucleotide primers corresponding to the third and seventh transmembrane domains of the secretin/calcitonin/parathyroid hormone family of GPCRs. Using this cDNA fragment, they then cloned the VIP2 receptor which encodes 437 amino acids protein, from a rat olfactory bulb cDNA library. [28].

In another study, Inagaki and colleagues isolated a subtype of the PACAP receptor [32]. They began by isolating the cDNA of putative receptors by RT-PCR with primers which were selected from a region of homology among GPCRs of the VIP/glucagon/secretin family from rat pancreatic islet RNA. Using the cDNA of the putative receptors as a probe, a novel receptor was then cloned by hybridization from a MIN6 cDNA library which was generated from a mouse insulin-secreting cell line. This receptor was designated PACAPR-3.

Usdin and colleagues also later reported cloning of the same receptor from a rat cerebral cortex cDNA library [33], and Rawlings and colleagues adopted the term PVR3 [30]. Other groups also independently reported the existence of the same novel receptor within a short period [34], and it was subsequently termed the VPAC2-Receptor by the International Union of Pharmacology [24] (Table 2).

The human VPAC2-Receptor cloned from a human placenta cDNA library using a combination of techniques including PCR with primer corresponding to conserved regions in the first-intracellular loop and the seventh transmembrane domains of the secretin, PTH, glucagon, and VIP receptors, and cDNA licrary screening. This receptor comprises 438 amino acids and possesses 87% sequence identity with rat VPAC2-R [35].

### Distribution of PACAP and its receptors in the CNS

A few years after isolation of PACAP, the distribution of PACAP and its receptor has been thoroughly studied in the rat brain.

In the rat barin, the precursor of the PACAP mRNA is present in the olfactory bulb, the cingulate cortex and the cortex extract in cerebral cortex, the hippocampus, the thalamus, and the hypothalamus, and the highest concentrations of PACAP are located in hypothalamic area [36, 37].

The PAC1-Receptor mRNA are observed over wide area such as the olfactory bulb, the cingulate cortex, piriform cortex, the dentate gyrus of the hippocampus, the paraventricular nucleus, the ventromedial nucleus and the supraoptic nucleus of the hypothalamus, the cerebellum, the lateral paragigantocellular, the pontine nuclei and the vagal complex of the brainstem, and the spinal cord [38–41].

In these areas, the localization of PAC1-R mRNA correlates well with the distribution of typeI (PACAP-specific) binding sites which examined by autoradiography [9, 41–44].

The VPAC1-R mRNA is present mainly in the cerebral cortex and the hippocampus, whereas VPAC2-R mRNA is predominantly expressed in the central nucleus of amygdala, the hippocampus, thalamus, hypothalamus, and the pontine nuclei of brainstem [27, 33, 45].

The distribution of TypeII (PACAP/VIP) binding sites is more restricted than that of typeI binding sites, and are located in the olfactory bulb, the cerebral cortex, the dentate gyrus of the hippocampus, thalamus, the locus coeruleus of the pons, area postrema, and the spinal cord [46–50]. For more details on distribution, see the excellent review in [1].

As described above, the distribution of PACAP and its receptor is well established in the rodents, little information is available regarding its distribution of human brain. Concentrations of pituitary adenylate cyclase activating polypeptide (PACAP) in microdissected human brain regions were measured by radioimmunoassay. The highest concentrations of PACAP were observed in the dorsal vagal complex, the bed nucleus of the stria terminalis, the median eminence-pituitary stalk, and in the periventricular and paraventricular hypothalamic nuclei. In addition, they were found in some hypothalamic (supraoptic and ventromedial), preoptic and brainstem nuclei. High concentrations were also measured in the septum pellucidum, periaqueductal and spinal gray matters, the motor facial, and in the spinal nucleus of the trigeminal nerve [51].

About the PACAP receptors, the distribution in the human cerebellum during development was reported [52].

In human fetuses, PAC1-R mRNA is associated with the external granule cell layer (EGL), a germinative neuroepithelium, and with the internal granule cell layer. The distribution pattern of VPAC1-R mRNA during development was very similar to that of PAC1-R mRNA, whereas VPAC2-R mRNA was visualized only in adults. The distribution of ^125^I-PACAP27 binding sites was consistent with that of PAC1-R and VPAC1-R mRNA [52].

## Conclusion

This review introduce an overview of the history of the discovery of PACAP and its three receptors. PACAP and its receptors are involved in diverse biological functions. An understanding of their signaling pathways may lead to the development of new therapeutic drugs.
